# Prevalence of diarrheal diseases and associated factors among under-five children in Dale District, Sidama zone, Southern Ethiopia: a cross-sectional study

**DOI:** 10.1186/s12889-019-7579-2

**Published:** 2019-09-06

**Authors:** Behailu Melese, Wondimagegn Paulos, Feleke Hailemichael Astawesegn, Temesgen Bati Gelgelu

**Affiliations:** 1 Hamlin Fistula Center Yirgalem, Yirgalem, Ethiopia; 2 School of Public Health, College of Health Sciences and Medicine, Wolaita Sodo University, Wolaita Sodo, Ethiopia; 3 School of Public Health, College of Health Sciences and Medicine, Hawassa University, Hawassa, Ethiopia

**Keywords:** Dale District, Diarrhoeal diseases, Under-five children

## Abstract

**Background:**

Globally childhood diarrhoeal diseases continue to be the second leading cause of death, while in Ethiopia it kills half-million under-five children each year. Sanitation, unsafe water and personal hygiene are responsible for 90% of the occurrence. Thus, this study aimed to assess the prevalence and associated factors of diarrheal diseases among under-five children in Dale District, Sidama Zone, Southern Ethiopia.

**Methods:**

A community-based cross-sectional study was conducted. A face to face interview using a structured questionnaire and observation checklist was used. A total of 546 households with at least one under-five children were selected using simple random sampling techniques. The data entry and cleaning were performed using Epidemiological information software (EPI Info) 3.5.1 and then exported to Statistical Package for Social Science (SPSS) version 16.0 for analysis. Frequencies and proportions were computed as descriptive analysis. Initially using bivariate analysis a crude association between the independent and dependent variables was investigated. Then, those variables with *p*-value ≤0.25 were included in multivariable analysis to determine the predictor variables for the outcome variables. Finally, further analyses were carried out using multivariable analysis at a significance level of p-value ≤0.05.

**Results:**

A total of 537 children under the age of 5 years were included. The 2 weeks prevalence of diarrhea among children under the age of 5 years was 13.6, 95% CI (10.7, 16.5%). Educational level [AOR: 3.97, 95% CI (1.60, 8.916)], age of indexed child [AOR: 12.18, 95% CI (1.78, 83.30)], nutritional status [AOR: 6.41, 95% CI (2.47, 16.77.)], hand washing method [AOR, 3.10, 95% CI (1.10, 8.67)], hand washing after latrine [AOR: 2.73, 95% CI (1.05, 6.56)], refuse disposal method [AOR, 3.23, 95% CI (1.37, 7.60)] and housing floor material [AOR: 3.22, 95% CI (1.16, 8.91] were significantly associated with the occurrence of childhood diarrheal diseases.

**Conclusion:**

Childhood diarrhea remains the commonest health problem in the study area. The findings have important policy implications for childhood diarrhoeal disease intervention programs. Thus, activities focusing on proper handwashing techniques at all appropriate times, proper refuse disposal, improving nutrition and better childcare also highly recommended.

## Background

According to world health organization (WHO), Diarrhea is characterized by three or more loose or liquid stools per day due to abnormally high fluid content of stool Or an abnormal increase in daily stool fluidity, frequency, and volume from what is considered normal for an individual [1]. Diarrheal diseases have been a major public health concern of low-income countries leading to high morbidity and mortality among under-five children [2].

Each year, an estimated 2.5 billion cases of diarrhea occur among children under the age of five, and estimates suggest that overall incidence has remained relatively stable over the past two decades [1]. Diarrheal diseases account for 1 in 9 or 9% of child deaths worldwide, making diarrhea the second leading cause of death among children under the age of five [3, 4] Globally, from all causes of child deaths that occurs daily,diarrheal diseases accounts 15%/ more than 1600 children deaths under 5 years of age [5, 6].

In Africa and South Asia more than four-fifths of all under-five deaths (82%) caused by diarrheal diseases [7]. From all deaths worldwide, about half of them due to pneumonia and diarrhea occur in just five most poor countries: namely India, Nigeria, the Democratic Republic of Congo, Pakistan, and Ethiopia [1]. Although Ethiopia has already achieved remarkable progress in reducing under-five mortality in the last decades, diarrheal diseases are still a common problem, Studies done in different parts of Ethiopia had shown that diarrhea is still a major public health problem. According to Ethiopia Demographic and Health Surveys (EDHS) of 2016, 12% of children under age five had diarrhea [8]. Similarly, this report showed that in southern nations nationalities and people region 13.9% of children under the age five have had diarrhea [8]. Particularly, in Sidama Zone Shebedino district the 2 weeks prevalence of diarrhea was (19.6%) which is higher than that of regional and national prevalence [9].

Therefore, though the factors that contribute to the occurrence of diarrheal diseases among children under the age of 5 years is complex, the relative contribution of socioeconomic, environmental, and behavioral factors should not be underestimated [8]. Despite the emphasis given by the Ethiopian ministry of health and the respective regional health offices to improve child health still, many children are dying due to easily preventable and treatable diarrheal disease in Ethiopia. So, this study aimed to assess the prevalence and associated factors of diarrheal diseases among children under the age of 5 years. Knowing this information would help to design appropriate intervention strategies to avert diarrheal disease. It will also provide information for program providers and managers to address the gap. Furthermore, the findings obtained from this study will provide baseline information for further researchers.

## Methods

### Study design and area

A community-based cross-sectional study was conducted from October 1–25/2017 in Dale district, Southern Ethiopia. The District has 3 urban and 36 rural kebeles. Agriculture is the main livelihood of the population with Enset, Teff, maize, millet, barley, and legumes being the main crops cultivated in the district. The district has an estimated 272,212 total populations of these 42,492 are under-five children and 50, 113 households. In the district, there are one hospital, 10 health centers, 36 health posts & three private health institutions that provide preventive and curative services to the community. The potential health services coverage of the Woreda was 70%, but according to the annual report of Dale District health office, in 2015/16 Pneumonia, Helminthiasis, Diarrheal diseases, Acute Respiratory Infection and Malnutrition were the top five diseases of under-five morbidity in the district [10]. But there is no study documented on the prevalence and factors associated with diarrheal diseases in the district. Therefore, this study aimed to assess the prevalence and associated factors of diarrheal diseases among under-five children in Dale District, Sidama Zone, Southern Ethiopia.

### Study and source population

Source population were all under-five children living in the Dale district and the study population were randomly selected households (HHs) with at least one under-five children.

### Sample size calculation

The sample size was calculated using two population proportion formula with the following assumptions; 95% level of confidence, 85% power of the test, design effect of 2 and 10% non-response rate. (*P*_1_ = 76.48%) the proportion of poor Waste Disposal among cases and (*P*_2_ = 59%) proportion of poor Waste Disposal among controls [11]. In the meantime, the sample size was calculated by the statical program of Epi Info 3.5.3. Then the final sample size was 546.

### Sampling technique

In the first stage, one urban kebele and ten rural kebeles were selected using simple random sampling from 3 urban and 36 rural Kebeles after stratifying the kebeles based on their residence. In the second stage after proportionally allocating a total sample size of 546 HHs to each selected kebeles, then HHs with at least one under-five children were randomly selected. In the meantime, we used the lottery method to include one child for those HHs with more than one under-five children. The first household was selected randomly at the center of the kebele and the subsequent households were selected systematically to the left side of each household and sampling interval used for selected kebeles. When the selected households had no under-five children, the next neighborhood household was replaced.

### Data collection tools and methods

The structured questionnaire was developed after reviewing relevant literature to include all the possible variables that address the objectives of the study. The questionnaire contains independent variables such as socioeconomic and demographic characteristics, demographic and health characteristics of the indexed children, behavioral factors and environmental sanitation factors. The questionnaire first prepared in English and then translated to local language Sidama and back-translated to English to maintain the consistency of the contents of the instrument using language expert. Face to face interviews was conducted by eight Diploma holders and supervised by two Degree holders to collect data about the above variables, besides this observational checklist was also used. Anthropometric indicators for children were collected based on a comparison of weight-for-age with a reference population of well-nourished children. Based on WHO Multicentre Growth Reference Study weight-for-age of the children was computed, then nutritional status determined. Consequently, those children whose weight-for-age found below minus two standard deviations (− 2 SD) and minus three standard deviations (− 3 SD) from the median of the reference population were categorized as moderately and severely malnourished respectively [12].

### Data quality assurance

To assure the quality of data Diploma holder data collectors and Degree holder supervisors with health background were assigned. The training was provided for 2 days about interview technique, how to maintain quality of data, and ethical issue for data collectors and supervisors.

A pre-test was done on 5% of the sample size in three none selected kebeles of (2 rural and 1urban) the Dale District. Necessary corrections were made based on the finding of a pre-test. Completed questionnaires were checked for its consistency and completeness daily.

### Data management and analysis procedure

The data entry and cleaning performed using EPI Info version 3.5.1 Statistical package and then exported to SPSS version 16.0 for analysis. Frequencies and proportions were computed as descriptive analysis. Initially using bivariate analysis a crude association between the independent and dependent variables was investigated. Then, those variables with *p*-value ≤0.25 were included in multivariable analysis to determine the predictor variables for the outcome variables. Before further analyses were carried out using multivariable analysis at a significance level of p-value ≤0.05, multicollinearity was checked among selected independent variables using the variance inflation factor and none was found. Additionally, Goodness of fit of the final model was checked by Hosmer and Lemeshow and was found fit.

### Operational definition

#### Diarrhea

Is defined as having three or more loose or watery stools in 24 h, as reported by the mother/caretaker of the child in the past 2 weeks before data collection [1].

#### Index child

Refers to a child that will be included in the study from a household to have information on the demographic and health characteristics [13].

#### Proper disposal of wastes/refuse

This is a way of disposal of refuses that included burning, burying in a pit or storing in a container and disposing of in a designed site, whereas disposing of in open fields considered as un proper disposal method [14].

#### Improved water source

Drinking-water sources protected from outside contamination. This includes protected spring, protected dug well and piped water [13].

## Results

### Socio-economic and demographic characteristics of study participant

A total of 537 households with children under the age of 5 years were included in this study with a response rate of 98.35%. Out of the total respondents, 504 (93.9%) interviews were conducted with mothers of children under-5 years of age and the rest with caretakers. From a total, 478 (89%) of the respondents were from the rural resident. The Mean age of respondents was 27.86 (±4.5 SD) years. Two hundred eighty-nine (53.8%) respondents did not attend formal education. The majority of respondents 488 (90.9%) were housewives. The mean family size of the study participants was 4.67 (±1.48 SD) (Table 1).

**Table 1:** Socioeconomic and demographic characteristics of study participants in Dale District, Southern Ethiopia, October 1–25/2017

Variables	Category	Frequency(n)	Percent (%)
Residence	Rural	478	89.0
	Urban	59	11.0
Number of household members	< 5	292	54.4
	≥5	245	45.6
Number of under-five children	One	487	90.7
	Above one	50	9.3
Age of the mother/caretaker	15–24	113	21.0
	25–34	379	70.6
	≥35	45	8.4
Marital status of the mother/caretaker	Married	523	97.4
	Others	19	2.6
Religion of parents	Protestant	509	94.8
	Others	28	5.2
Educational status of mother/caretaker	No formal education	289	53.8
	Formal education	142	26.4
Occupation of the mother/caretaker	Housewife	488	90.9
	Others	49	9. 1
Age of the child’s father	15–24	12	2.2
	25–34	313	58.3
	≥35	212	39.5
The educational level of a child father	No formal education	249	46.4
	Formal education	99	18.4
Occupation of the child father	Farmer	381	70.9
	Government employer	47	8.8
	Others	35	6.5
Monthly family income in birr	< 500 Birr	200	37.2
	500–1000 Birr	228	42.5
	> 1000 Birr	109	20.3

### Demographic and health characteristics of indexed children

Of the total children, 301 (56.1%) were males. Regarding the age group, 259 (48.2%) were greater than 24 months, followed by age between 12 and 24 months which accounts for 157 (29.2%). The mean ages of indexed children were 23.4 (±13.9 SD) months. Three hundred ninety-three (96.1%), 423 (94.6%) and 439 (92.1%) of the children received the Rotavirus vaccine, the measles virus vaccine, and Vit A Supplementation respectively. Seventy-three (13.6%) of the children had experienced diarrhea in the 2 weeks preceding the study (Table 2).

**Table 2:** Demographic and health characteristics of indexed children in Dale District, Southern Ethiopia, October 1–25/2017

Variables	Category	Frequency(n)	Percent (%)
Sex of the indexed child	Male	301	56.1
	Female	236	43.9
Age of the indexed child in a month	Below 6 months	62	11.5
	B/w 6–11 months	59	11
	B/w 12-24 months	157	29.2
	Greater than 24 months	259	48.2
The birth interval of indexed child	1st child	163	30.4
	≤ 2 yrs	81	15.1
	2 yrs. -4 yrs	216	40.2
	≥ 4 years	77	14.3
Current Breast Feeding (BF) status of the indexed child	Exclusively breastfeed	71	13.2
	Partially breastfeed	363	67.6
	None breastfeed	103	19.2
The child started Supplementary/Complementary feeding	Yes	466	86.7
	No	71	13.3
Measles Vaccination status *n* = 447	Yes	423	94.6
	No	24	5.4
Vit.A Supplementation	Yes	439	92.1
	No	38	7.9
Rota virus immunization	yes	393	96.1
	No	16	3.9
Nutritional Status	Under nutrition	122	22.7
	Normal	415	77.3
Presence of diarrhea in the last 2 weeks	No	464	86.4
	Yes	73	13.6
The child was ever taken to Health institutions, *n* = 73	Yes	17	23.3
	No	56	76.7
Use of ORS to treat child, n = 73	Yes	35	47.9
	No	38	52.1

### Behavioral characteristics of respondents

Two hundred ninety-one (54.2%) mothers/caretakers wash their hands using soap/ash and water after visits of the latrine. Out of a total of 396 (73.7%), respondents reported that they practice hand washing before food preparation and 422 (78.6%) after latrine visit. From total respondents, 495 (92.2%) had a separate container for drinking water storage and 483 (89.9%) had cover for the drinking water container. Eighty-nine (16.6%) of respondents treat their drinking water at home (Table 3).

**Table 3:** Selected behavioral characteristics of respondents in Dale district, Southern Ethiopia, October 1–25/2017

Variables	Category	Frequency(n)	Percent(%)
Food child mostly receive	Cow’s milk	140	26.0
	Gruel	110	20.5
	Adults’ food	216	40.2
Separately preparation of child food	Yes	306	57.0
	No	160	29.8
Materials used to feed a child	Hand	287	53.4
	Bottle	54	10.1
	Spoon	196	36.5
Cover for drinking water storage container	Yes	483	89.9
	No	54	10.1
Separated container for drinking water	Yes	495	92.2
	No	42	7.8
Use latrine all the time	Yes	489	91.1
	No	48	8.9
Methods of washing hands	Water only	131	24.4
	Water and soap/ash	291	54.2
	Not washing hand	115	21.4
Hand washing after feeding the child	Yes	459	85.5
	No	78	14.5
Hand washing before feeding the child	Yes	396	73.7
	No	141	26.3
Hand washing after visiting the latrine	Yes	422	78.6
	No	115	21.4
Hand washing after cleaning a child’s bottom	Yes	338	62.9
	No	199	37.1
Home treatment of drinking water	Yes	89	16.6
	No	448	83.4
Age of child started supplementary food	Less than 6 month	131	24.4
	At 6 month	259	48.2
	Greater than 6 month	147	27.4
Time of initiation of BF during birth	Within 1 h	507	94.4
	After 1 h	30	5.6

### Environmental sanitation characteristics of households

Of total respondents, 275 (51.2%) had two and above separate rooms in the house. Out of the total respondents, 496 (92.4%) had a latrine. Two hundred twenty-nine (46.2%) of the households of respondents had a handwashing facility near to the latrine. From total 489 (91.1%) latrine facilities were privately owned. In 270(54.4%) respondents of the households, no feces observed around the latrine hole. Two hundred eighty-five (53.1%) of households disposed of refuse in open field, With regards to their drinking water source 130 (24.2%) respondents reported that they get from the unprotected water source. Out of a total, 265 (49.3%) had consumed 20-l water or less per day (Table 4).

**Table 4:** Environmental sanitation characteristics of households, in Dale district, Southern Ethiopia, October 1–25/2017

Variables	Category	Frequency(n)	Percent (%)
Floor of Housing	Mud	389	72.4
	Cement	148	27.6
Roof of Housing	Wood/ thatched	410	76.4
	Corrugated iron	127	23.6
Numbers of rooms	< 2 Rooms	262	48.8
	≥2 Rooms	275	51.2
Latrine availability	Yes	496	92.4
	No	41	7.6
Ownership of the latrine *n* = 496	Privately	489	91.1
	Shared	7	1.3
Types of toilet facility *n* = 496	^a^Improved	290	58.5
	^b^Unimproved	206	41.5
Availability of handwashing facility near to toilet *n* = 496	Yes	229	46.2
	No	267	53.8
Presence of feces in the latrine hole *n* = 496	Yes	226	45.6
	No	270	54.4
Presence of feces inside the compound	Yes	53	9.9
	No	484	90.1
Refuse disposal methods	In-pit	152	28.3
	Open field	285	53.1
	Garbage bin	11	2.0
	Burning	89	16.6
Source of drinking water	Protected spring/well water	253	47.1
	Unprotected spring/well water	130	24.2
	Pipe	154	28.7
Time to reach a water source	≤30 Minutes	461	85.8
	> 30 Minutes	76	14.2
Water consumption per day	≤20 lit	265	49.3
	> 20 lit	272	50.7
Houses shared with domestic animals	Yes	244	45.4
	No	293	54.6

^a^indicate types of toilet improved when the toilet is Pit latrine with slab

^b^indicate types of toilet unimproved when the toilet is Pit latrine without a slab or pour-flush latrine

### Prevalence of diarrhea

Seventy three (13.6%), 95% CI (10.7, 16.5%) of the children had experienced diarrhea in the 2 weeks preceding the study. Out of 73 children who had got diarrhea only 17 (23%) of them taken to a health institution. The study also showed that 38 (48%) of children with diarrhea received oral rehydration (ORS).

### Factors associated with childhood diarrheal disease

Variables in the bivariate analysis of socio-economic, environmental sanitation conditions, behavioral conditions and child characteristics with respect to childhood diarrhea; which were found at *p*-value ≤0.25 were further considered for multiple logistic regression analysis. Accordingly, mothers’/caregivers’ educational status, age of the child, nutritional status of child, hand washing practice of mother/caregiver after latrine visit, mothers/caregiver handwashing method, methods of refuse disposal and housing floor material were a candidate for multiple logistic regression (Table 5).

**Table 5:** Factors Associated with diarrheal disease among under-five children in Dale District, Southern Ethiopia, October 1–25/2017

Variables	Category	COR(95% C.I)	AOR(95% C.I)
The educational level of child-mother/caretaker	No formal education	2.7 (1.6–4.8)	3.97 (1.60,8.81)**
	Formal education	1	1
Age of the index child in a month	Below 6 months	1	1
	B/w 6–11 months	0.29 (0.09–0.93)	2.42 (0.25,24.03)
	B/w 12–24 months	2.5 (1.26–4.8)	12 (1.78, 83.18)**
	Greater than 24 months	1.4 (0.67–2.9)	2.74 (0.39,19.09)
Nutritional status	Under nourished	4.91 (2.9–8.23)	6.41 (2.47,16.77)**
	Normal	1	1
Hand washing practice	Not washing hand	5.1 (2.475–10.7)	3.10 (1.10,8.67)*
	Water only	6.8 (3.427–13.6)	6.41 (2.51,16.39)**
	Water and soap/ash	1	1
Washing hand before feeding a child	Yes	2.21 (1.329–3.7)	1.20 (0.51,2.81)
	No	1	1
Washing hand after visiting the latrine	Yes	5.45 (3.24–9.18)	2.73 (1.05,6.56)*
	No	1	1
Washing hand after cleaning the child’s bottom	Yes	4.60 (2.70–7.81)	1.88 (0.78,4.73)
	No	1	1
A separate container for drinking water storage	Yes	1	1
	No	5.33 (2.71–0.48)	2.67 (0.81,8.75)
Consumption of water per day	≤ 20 Liters	2.7 (1.60–4.61)	1.27 (0.46, 3.47)
	Above 20 Litres	1	1
Housing floor material	Mud	2.66 (1.33–5.35)	3.22 (1.16, 8.91)*
	Cement	1	1
Source of drinking water	Safe	1	1
	Unsafe	1.54 (1.12–1.99)	2.14 (0.96, 4.85)
Refuse disposal methods	Proper way	1	1
	Improper way	2.65 (1.53–4.57)	3.23 (1.37, 7.60)**

*COR* crude odds ratio and *AOR* adjusted odds ratio

*P ≤ 0.05, **P ≤ 0.01 was considered statistically significant

Children whose mother/caretakers with no formal education had 3.97 times [AOR: 3.97, 95% CI (1.60, 8.91)], higher odds of developing diarrhea when compared to those mothers/caretakers with formal education. Children within the age group of 12–24 months had more than 12 times [AOR: 12.18, 95% CI (1.78, 83.30)] higher odds of developing diarrhea than those aged below 6 months. Under-nourished children had 6.41 times [AOR, 6.41, 95% CI (2.47, 16.77)] higher odds of developing diarrhea when compared to those with normal. Children of mothers/caretakers who had no habit of washing their hand after latrine visit had 2.73 times [AOR: 2.73, 95% CI: (1.05, 6.56)] higher odds of developing diarrhea when their hands with soap. Children of mothers/caretakers who had no habit of washing their hands with soap after latrine visit had 3.1 times [AOR, 3.10, 95% CI, (1.10, 8.67)] higher odds of developing diarrhea when compared with children whose mothers/ caretakers washed their hands.

Children whose family disposes of refuse improper way had above 3 times [AOR, 3.23, 95% CI, (1.37, 7.60)] higher odds of developing diarrhea than those families properly dispose of refusal. Children whose house had a dwelling with the Mud floor had 3.22 times [AOR: 3.22, 95% CI (1.16, 8.91)] higher odds of developing diarrhea when compared to those households had a dwelling with cement (Table 5).

## Discussion

In this study, the 2 weeks’ prevalence of diarrhea among under-five children is in line with the EDHS 2016 national and regional report [8]. But it is lower than other several similar studies conducted in Ethiopia: Eastern Ethiopia (22.5%) [14], Arbaminch rural community (31%) [13], Enderta district, Northern Ethiopia (35.6%) [15] and Enemy district (18.6%) [16]. similarly, this report is lower than the reports of studies conducted in Northwestern Burundi (32.6%) [17], West Banga, India (22.36%) [18] And in Kushtia, Bangladesh (44.5%) [19]. The lower prevalence report of our study may be due to improved sanitation and hygiene practices of the respondents. On the other hand, this study reports a higher prevalence than studies conducted in the Gambia, 7.7% [20] and in India, (5%) [21].

This study reports odds of developing diarrheal diseases among children under the age of 5 years were 3.97 times higher among children of mothers/caretakers who had not attended formal education compared with the odds of diarrheal diseases among children whose mothers had attended formal education. This is in line with other studies finding conducted in Arbamich district, Southern Ethiopia [13], Benishangul Gumuz region [11] and Sheko District, South West Ethiopia [22]. Similarily findings from India also showed a relatively higher prevalence of diarrhea among children whose mothers had no education [23]. This might be an educated mother’s will have a positive influence on hygienic practice, child feeding, weaning.

The odds of developing diarrheal diseases among children under the age of 5 years were 12 times higher among children aged from 12 to 24 months compared with the odds of diarrheal diseases among children aged below 6 months. This finding is in agreement with other studies conducted in a different part of Ethiopia [13, 14, 22, 24]. Similarly, studies in Bangladesh and India had also shown higher odds of diarrhea in children aged from 12 to 23 months [18]. This might be due to the combined effects of declining levels of antibodies acquired from the mother, lack of active immunity in the infant, the introduction of food that may be contaminated with fecal bacteria, and direct contact with human or animal feces [25]. In addition to this, at 12 to 23 months infants are either crawling or walking and as such, can easily pick dirt or other contaminated objects [11, 26].

Similarly, developing diarrheal diseases is 6.41 times higher among undernourished children compared with normal children. This is in line with a study conducted in Ethiopia, India, and Indonesia [9, 21, 27, 28]. This might be due to malnutrition weakened the immune system leads to an increased susceptibility to diarrheal diseases and other infections [21, 23].

Furthermore, having diarrheal diseases among children under the age of 5 years was 2.73 times higher among children of whose mothers/caretakers not practiced hand washing using soap/ash after toilet visit compared with the odds of diarrheal diseases among children whose mothers/caretakers practice hand washing using soap/ash after latrine visit. This finding is in agreement with studies conducted in Shekko and Dejen district in Ethiopia [22, 29]. In agreement with this study, another study also reported as handwashing with soap particularly before eating, preparing food and feeding children and after using the toilet can considerably reduce the risk of diarrhoea [30]. Handwashing with water and soap is the most effective health intervention for reducing the incidence of diarrhea in children under the age of five [25]. This is can be explained by Soap breaks down grease and dirt that carry germs and disease-causing pathogens [30].

According to the results of this study, the odds of having diarrheal diseases among children under the age of 5 years were 3.23 times higher among children from households that disposes of refuse improperly compared with the odds of diarrheal diseases among children from households which properly disposes of refuse. This is in agreement with studies conducted in Ethiopia Enemy District [30], Shekko district [22] and Eastern Ethiopia [14]. This could be due to refuse may contain different pathogens which can cause diarrheal diseases and creates a suitable site for insects breeding. So, improper refuse disposal increases the chance of contact of insect vectors from refuse to food items worsening sanitation and hygiene of family.

The odds of developing diarrheal diseases among children under the age of 5 years were 3.22 times higher among children from households who had a dwelling with the Mud floor compared with the odds of diarrheal diseases among children whose household dwelling with cement floor. This could be due to housing floor material that might be related to a clean home environment [31]. In addition to this, a clean home environment is critical for reducing the transmission of pathogens that cause diarrheal diseases. The house floor material may significantly affect the occurrence of childhood diarrhea [7]. For readers as a limitation, there might be reporting bias during face to face interviews which is tried to be minimized by giving pre-data collection training for data collectors. There might also recall bias on vaccination status, but we tried to limit recall bias by checking the card. There may be a difference among respondents about the definition of diarrhea which was considered during the design, and the definition of diarrhea was explained. In addition to the above limitations, cause and effect cannot be ascertained since it is a cross-sectional study.

## Conclusion

The 2 weeks’ prevalence of diarrhea among children under the age of 5 years was 13.6%. The independent variables that were found to be associated with diarrheal diseases among children under the age of 5 years were the educational status of mother/caretakers, age of the indexed child, nutritional status of the indexed child, methods of hand washing, refuse disposal methods and housing floor material. These findings have important policy implications for childhood diarrhoeal disease intervention programs. Government sectors and partners working in collaboration to implement an intervention program focusing on the community to practice proper handwashing techniques at all appropriate times, proper refuse disposal. Improving nutrition and better childcare also highly recommended.

## Data Availability

The datasets used and/or analyzed during this study are available from the corresponding author and provided on a reasonable request.

## Abbreviations

BF: Breast Feeding
CI: Confidence Interval
EDHS: Ethiopia Demographic and Health Survey
Epi info: Epidemiological information software
HH: House Hold
OR: Odd Ratio
ORS: Oral Rehydration Solution
SPSS: Statistical Package for Social Science
UNICEF: United Nations International Children’s Emergency Fund
WHO: World Health Organization
